# Seasonal Variation in Bright Daylight Exposure, Mood and Behavior among a Group of Office Workers in Sweden

**DOI:** 10.5334/jcr.153

**Published:** 2018-02-21

**Authors:** Mathias Adamsson, Thorbjörn Laike, Takeshi Morita

**Affiliations:** 1 Jönköping University, School of Engineering, SE; 2 Lund University, SE; 3 Fukuoka Women’s University, JP

**Keywords:** Light exposure, Psychological wellbeing, Lighting conditions, Circannual, Sleep-wake behavior, High latitude

## Abstract

The purpose of the study was to investigate seasonal variation in mood and behavior among a group of office workers in Sweden (56°N). Thirty subjects participated in this longitudinal study. The subjects kept a weekly log that included questionnaires for ratings of psychological wellbeing and daily sleep-activity diaries where they also noted time spent outdoors. The lighting conditions in the offices were subjectively evaluated during one day, five times over the year. There was a seasonal variation in positive affect and in sleep-activity behavior. Across the year, there was a large variation in the total time spent outdoors in daylight. The subjects reported seasonal variation concerning the pleasantness, variation and strength of the light in the offices and regarding the visibility in the rooms. Finally, the subjects spent most of their time indoors, relying on artificial lighting, which demonstrates the importance of the lighting quality in indoor environments.

## Introduction

Seasonal variation in various aspects of human physiology, neuroendocrine function and behavior is quite common [1,2,3,4,5,6,7,8,9,10]. Grimaldi et al. [11] reported that 85 per cent of the subjects in a study conducted in Finland experienced variations in mood and behavior. The results from a study using the Seasonal Pattern Assessment Questionnaire (SPAQ) to investigate the prevalence of winter depression symptoms in Sweden showed that 53.2 per cent of the subjects experienced seasonal variation in depressed mood and lack of energy and 19.3 per cent of the respondents reported this to be a problem that affected daily life [12]. In line with other results, seasonal problems were more common in women than in men [12,13]. Moreover, Kasper et al. [1] found that only eight percent of the respondents in an American survey reported no changes in mood and behavior.

Light could be one important factor in regulating the different systems. Since there is a difference in the length of day and thus the amount of radiation will differ at different time of the year in countries far from the equator this may be seen in the overt behavior of the individual.

There is evidence that light has an impact on the mood. For example, studies investigating light treatment provided by LED (Light Emitting Diode) light sources have shown positive results after repeated morning exposures to monochromatic short-wavelength light radiation or polychromatic light enriched in the short-wavelength part of the spectrum [14,15,16,17].

Other studies have demonstrated that bright light therapy administrated either in the morning, or during the midday or evening, or both in the morning and evening have beneficial effects. However, three large studies [18,19,20] have shown a higher efficacy of bright light treatment in the morning in comparison to light treatment in the evening or a combination of morning and evening exposures. Conversely, Wirz-Justice et al. [21] found no effect of the timing of bright light treatment and according to a meta-analysis of the efficacy of light treatment with medium light intensity, a combination of morning and evening exposure showed a higher efficacy than a single pulse in the morning, midday or evening [22].

There have been inconsistent results from earlier and more recent studies investigating the therapeutic effect of bright light exposure in healthy subjects. Partonen and Lönnqvist [23] reported that repeated exposures to an hour of bright light per day in the winter, at home or in the workplace, had a positive effect on mood and improved vitality in a healthy population and subsequently had a positive effect on health-related quality of life. Furthermore, Goel and Etwaroo [24] showed that repeated exposures to a 30-minute pulse of bright light with an intensity of 10,000 lux, administered in the evening, decreased depressive symptoms and improved mood both in depressed and non-depressed university students after 15–30 minutes. Other studies have not shown any effect of bright light treatment in healthy populations [25,26,27] or reported negative, adverse effects, for example eye irritation and a deterioration of mood, possibly as a consequence of “hyperarousal” [28,29,30].

It is clear that there is an ambiguity between the results which may be explained by differences in the methods used, for example type of psychological instruments. Other explanations for discrepancies between the findings may be connected to differences regarding light exposure (i.e. spectral quality, intensity and exposure pattern). Furthermore, models for describing the effects of lighting conditions on psychological wellbeing are at an early stage of development and this process appear to be complex involving both physiological and psychological aspects [31,32,33,34,35].

There are also studies that relate the seasonal differences with the amount of light it is possible to receive. In a cross-cultural study, Küller et al. [36] observed a noticeable seasonal variation in mood in Sweden and the UK. In contrast, the mood curve showed a more even profile during the year in Saudi Arabia and Argentina. Additionally, they found a relationship between the lighting conditions in the office and mood which showed that higher mood was reported when the lighting was experienced as “just right” but “too much” or “too little” light was associated with a lower rating of mood. Persson et al. [37] demonstrated a seasonal variation in stress, with higher levels experienced in the early afternoon during the winter and early spring for a group of Swedish subjects that rated their subjective health and stress during one workday every month across the year.

Studies conducted at different latitudes (30–52°) on the northern hemisphere have shown large seasonal differences in the daily light radiation to which people normally are exposed [8,38,39,40,41,42].

Espiritu et al. [43] reported a negative relationship between illumination, depression score and atypical depressive symptoms for a group of subjects living in San Diego (32 N). In a longitudinal study of subjects living in Rochester (44 N) and San Diego (32 N), Park et al. [3] found a seasonal variation in mood in Rochester but not in San Diego. Hubalek et al. [44] did not find any relationship between daily light exposure and mood or any influence of SAD (Seasonal Affective Disorder) scores on daily light exposure in a study of office workers in Switzerland (47 North, 8 East). However, they found a positive correlation between self-reported sleep quality and higher daily light exposure.

Several field studies, carried out in real living and working environments, have examined and compared different parameters regarding daily and seasonal light exposure in people suffering from seasonal variations in mood and people with no such problems. Eastman [45] showed that a group of SAD subjects, living in the Chicago area, received more than twice as much bright light (i.e. time spent in daylight outdoors) in the summer in comparison to the winter. In addition, the perceived skeleton period, a parameter defined as the time period between first and last outdoor exposure, was 4–5 times longer in the summer than in the winter. The results showed that the lengthening of the perceived skeleton period was asymmetrical with larger seasonal difference in the evening. Moreover, studies have shown that both people suffering from problems due to seasonal change in mood and vegetative behavior and healthy controls receive more light in the summer than during the winter [46,47,48]. In addition, findings have indicated a possible effect of photoperiod and amplitude difference in light exposure (i.e. difference between light exposure in the summer and in the winter) on mood [46,48]. In a group of mildly seasonal participants, aan het Rot et al. [49] found a positive relationship between the duration of bright light (i.e. light exposure exceeding 1000 lux), mood and positive social interaction irrespective of season. An above average exposure to bright light was related with less quarrelsome and more agreeable behaviors. Furthermore, Grandner et al. [50] found that a higher daily light exposure was correlated with more positive ratings of quality of life and a better social and emotional functioning in a sample of post-menopausal women. The authors also reported that morning light was related to these factors and was a stronger predictor of the benefits of light than mean daily light exposure.

In a longitudinal field study conducted in the Netherlands, Smolders et al. [51] used ambulatory measuring equipment to record daily light exposure. The results showed a positive relationship between the subjects’ experience of vitality and luminous exposure, especially during winter mornings indicating acute alerting effect of lighting radiation also during daytime. Moreover, Kaida et al. [52,53] showed that a 30-minute exposure to natural bright light during lunch had an immediate positive effect on mood during the exposure and had an increasing effect on arousal that lasted for one hour after the exposure. In a field setting, Wilhelm et al. [54] compared the effect of different light intensities; 500 lux, 1,000 lux, and 2,500 lux on objectively measured alertness, subjective ratings of alertness and mental well-being during daytime work. They only found a difference in the subjective measures between the two extremes. In a study carried out in Antarctica, Corbett et al. [55] reported an improvement in cognitive performance after exposing subjects to an hour of bright light in the morning during the winter.

Studies comprising general and population samples have investigated the occurrence of seasonal differences in various sleep characteristics. For example, findings have shown seasonal variations in bedtime [56] and time of awakening [57], sleep onset [8,58] and sleep duration [8,12,59]. In a study conducted in Norway (69°39’N) and Ghana (5°32’N), these results were confirmed for some, but not all the above-mentioned parameters [58]. On the other hand, Park et al. [3], did not find any seasonal variations regarding sleep onset time, time in bed, sleep latency, total sleep time and number of awakenings. Moreover, no seasonal difference was found concerning measures of activity. The contradictions of the results may be explained by differences in methodology, for example sample characteristics and method of data collection.

Taken together, the literature shows that light has a major influence on human physiology and consequently has an effect on psychological processes. However, the relative impact seems to vary depending on temporal, quantitative and qualitative aspects. Studies using different instruments for measuring exposing light radiation have shown seasonal variations as well as differences between people living on different latitudes. However, few studies have been conducted at latitudes above 50°N.

The aim of the present study was to report on the prevalence of seasonal variations in mood and behavior among a group of Swedish office workers living on latitude of 56°N with large variation in photoperiod across the year. Another aim of the study was to investigate the daily and seasonal pattern of bright daylight exposure outdoors, which includes much short-wavelength radiation at a large intensity, and in what way the subjects were exposed to light radiation from various light sources.

In line with results demonstrating photoperiod as the primary factor influencing seasonal variation in mood and behavior, it was hypothesized that the participants would report more positive ratings of mood during the summer than in the autumn, winter and spring. Moreover, a seasonal variation in exposure to bright daylight outdoors as well as a seasonal difference in the exposure pattern was expected as a consequence of changes in the length of the photoperiod but also due to annual variations in temperature which should have an impact on the amount of time spent outdoors during the various seasons.

In addition, a seasonal difference in the sleep-activity behavior was expected with later bedtimes during the summer in comparison to the other seasons. Due to seasonal difference in total daily solar radiance and a documented preference for daylight it was finally hypothesized that there would be a seasonal variation regarding the subjective evaluation of the lit environment in the offices with higher ratings during summer in comparison to the winter [60,61,62,63].

## Methods

### Subjects

The study was conducted in the south of Sweden (N 56°). 20 women (mean age = 42.6 years, SD = 9.98 years, range 24–61 years) and 10 men (mean age = 45.2 years, SD = 14.7 years, range 21–64 years) participated in the study. 12.5 per cent of the participants were morning type individuals and 28.1 per cent had an evening preference.

The subjects gave informed consent after attending a lecture about non-image forming effects of light held by the authors and where an explanation of the purpose, described as general environmental impact, and a general description of the procedure of the study was given. Moreover, the subjects were informed that participation in the study was voluntary and that they could withdraw at any time. Collection of data was confidential and the data was treated in a way that made identification of individual persons impossible.

The subjects worked in office environments during the daytime. The workweek included 36–40 h during weekdays. They started work between 07:00 and 08:30 and ended work between 15:00 and 18:30.

### Settings

The workplaces were located in private offices or small office landscapes with 3–6 desks. The office environments were mainly lit by localized lighting from ceiling-suspended luminaires with direct/indirect or indirect light distributions. The luminaires in the offices were equipped with fluorescent and compact fluorescent lamps and had a correlated color temperature between 3,000 K and 3,500 K. Most luminaires provided individual dimming control. Some subjects had access to additional task light. Moreover, downlights and wall luminaires equipped with compact fluorescent lamps were also used for illuminating the office environments.

All offices except one had windows and the subjects’ workplace were located on average 1.7 m (SD = 1.1 m, range = 1.1 m–6.8 m) from a side-window. Internal venetian blinds admitted manual control of the incoming daylight. The private offices had one or two side-windows and the small landscapes had three to twelve side-windows. Four of the offices had windows oriented towards north and four had windows facing northeast. Furthermore, two offices had windows facing west. Four of the offices had windows facing southeast and six offices had windows oriented towards southwest. In four of the offices the windows faced northwest. Glazed indoor partitions allowed some borrowed light to reach the offices from neighboring rooms. Table 1 shows climatic data for the location of the study. Table 2 shows mean ambient room temperature in the offices across the year.

**Table 1:** Climatic conditions for the location where the study was carried out [64].

Month	Hours of sunshine (h)	Global radiation (kWh/m^2^)	Days with rainfall/snowfall (number of days)	Mean air temperature (°C)

February	69	29.9	10	4.1
March	140	71.6	19	3.4
April	219	128.9	8	7.9
May	364	202.6	4	12.8
June	289	188.3	11	16.0
July	312	189.8	7	18.8
August	168	114	20	17.3
September	159	88.4	11	13.3
October	112	47.3	22	9.4
November	40	14.8	20	5.5
December	28	8.8	16	2.1
January	39	12.1	14	0.0

**Table 2:** Mean ambient room temperature across the year. Measurements were conducted with a calibrated Testo 720 digital temperature meter (Testo Se&Co. KGaA, Lenzkirch, Germany) in the morning and afternoon at five times (in February/March, in April/May, in June/July, in September/October, and in December/January).

Parameter		Feb/Mar	Apr/May	Jun/Jul	Sep/Oct	Dec/Jan

Room temperature	Mean (°C)	22.7	22.7	22.7	21.7	22.1
*N* = 30	SD (°C)	1.0	0.7	0.9	0.7	0.7

The information about the home environment is more limited. However, regarding the lighting, incandescent lamps and halogen lamps were generally used in the majority of the luminaires in the homes. Some luminaires in the home environments were equipped with integrated compact fluorescent lamps or fluorescent tubes.

### Dropouts

Two subjects dropped out at an early stage of the study, stating difficulties in following the study protocol as reason, and were not included in the data analysis.

Nine per cent of the questionnaires for rating of mood were returned unanswered. Furthermore, a total of eight percent of the data were missing from the assessments of sleep-activity behavior and concerning the subjective ratings of the lighting conditions in the office environments eight percent of the data were missing.

### Procedure

For one year, from February 2008 to January 2009, the subjects followed a monthly three-day experimental period, beginning at 12:00 on a Tuesday and ending 12:00 on Friday. On Tuesday morning they received a log which included questionnaires for daily ratings of psychological well-being and a daily sleep-activity diary. During one day in February, April, June, September and December the subjects assessed the lighting conditions in the offices in the morning and in the early afternoon. For collection of background data, the subjects completed a set of questionnaires at the beginning of the study. In order to allow the monthly collection of data across the year the experimental weeks of the subjects were planned with respect to their vacation. Before the start of the study the workplaces were visited by members of the research team and information concerning type of lighting fixtures, type of light sources, cardinal directions of the windows and distances between the normal working position and the windows in the offices, type of solar shading, type of controls for the artificial lighting and daylight, furniture and surface colors in the offices, orientation of computer monitors and connections between various rooms in the workplace were documented.

### Subjective Ratings

#### Affective state

PANAS, a form comprised of two ten-item mood scales for measuring positive affect, PA (α = 0.86–0.90) and negative affect, NA (α = 0.84–0.87), was used for ratings of psychological wellbeing at four times, 08:00; 12:00; 15:00; 20:00, per day. When added the twenty items result in a PA and a NA scale with a range between 10 and 50. These scales can vary in parallel and PA but not NA have been shown to display a circadian rhythm [65,66].

#### Subjective assessments of the office lighting

A questionnaire containing sixteen bipolar seven-grade scales was used for the subjective assessments of the lighting conditions in the offices [67,68]. Four orthogonal dimensions; pleasantness, or hedonic tone, brightness, or strength, flicker and light distribution, or variation, can be derived from the scales through factor analyze. Cronbach’s α is 0.84 and 0.82 for hedonic tone and strength respectively [69]. Based on data collected in the present study Cronbach’s α for variation is 0.52. In addition, the instrument provided a question regarding how well the lit environment supported the visual needs. The five seasonal assessments were made during one day at 08:00 and 15:00.

#### Sleep-activity behavior

A daily sleep-activity log was developed for the study, where the subjects graphically noted wake-up time, time when lights were turned off for sleep and work times. The daily log also contained a 24-hour graph, divided into ten-minute bins where the subjects noted the time they spent outdoors.

#### Background data

The subjects provided background data at one time, by answering a set of questionnaires during the first of the experimental periods. The background data included personal information, information concerning work schedules, habitual sleep and wake-up times, information about optical correction and color vision and estimation of habitual exposure to daylight during four time periods (early morning, morning, early afternoon and late afternoon). The subjects’ circadian preference was assessed by the following questions: I am a typical sort of person that likes to stay up late at night, I am a typical sort of person that likes to get up early in the morning, and I usually have difficulty falling asleep in the evening [68]. The three scales were graded as follows: Yes, I agree; I am not sure; No, I do not agree.

#### Light sources

The extent to which different light sources were used in the home environment was investigated by the use of a questionnaire that was developed for the study where the subjects indicated if a presented light source was used in most luminaires, in several luminaires, in a few luminaires or not at all. This questionnaire was answered at three occasions, in February, June and January. The questionnaire included the following light sources, depicted by photographs of the lamps: Incandescent lamps, halogen lamps, compact fluorescent integrated lamps, linear and compact fluorescent tubes. The form also permitted reports of other, not represented light sources that were used in the luminaires at home.

#### Seasonality

The subjects rated whether they were aware of higher subjective wellbeing during specific months of the year. The seasonal variation in mood was correspondingly measured by indicating the months when a lower mood was experienced and the strength of the emotion [36].

### Photoperiod length and time spent in bright daylight outdoors

Daily times for sunrise and sunset during the course of the year were calculated by using the sunrise/sunset calculator developed by National Research Council Canada [70]. The subjects’ daily reports of the timing of outdoor exposure were compared with those times in order to establish if the time were spent in daylight.

### Statistics

The Statistical Program for Social Sciences, SPSS, version 19, was used for the calculations. Seasonal variations in bright daylight exposure outside, mood, sleep-and activity behavior and subjective assessments of the lighting conditions in the offices were analyzed by means of ANOVA Repeated Measures. Additional analyses included seasonality as a between group factor.

Missing data from the assessments of positive and negative affect were replaced with individual seasonal mean for the corresponding time point. The seasons were divided as follows: spring included February, March and April, summer included May, June and July, autumn included August, September and October and winter included November, December and January. The procedure can be demonstrated by the following numerical example for one of the subjects. In May, the subject did not answer the PANAS schedule at 15.00 and 20.00 on the second day. The missing values were replaced by average PA at 15.00 and 20.00 during the summer, which were 31.6 and 26.9 respectively. Similarly, the missing values regarding NA were replaced with the average NA at 15.00 and 20.00 during the summer which were 15.6 and 13.9 respectively. Table 3 shows the PA and NA reported by the subject in May.

**Table 3:** An example for PANAS when missing values have been replaced by means.

	Day 1			Day 2				Day 3				Day 4	

Parameter	12.00	15.00	20.00	08.00	12.00	15.00	20.00	08.00	12.00	15.00	20.00	08.00	12.00
**(hh:mm)**													
PA	28	27	23	35	40	***31.6***	***26.9***	38	39	31	24	32	28
NA	17	24	21	16	23	***15.6***	***13.9***	16	14	18	15	19	14

Similarly, missing data regarding sleep and wake time were replaced with individual seasonal mean. Regarding subjective ratings of the lighting conditions in the working environments missing data were replaced by individual annual mean for the corresponding time point.

In addition, Pearson correlation coefficients were calculated to investigate interrelationship between different parameters.

## Results

### Retrospective Ratings of Seasonality

The retrospective ratings showed that a majority of the subjects experienced seasonal variation in subjective wellbeing and 46 per cent reported a noticeable or rather strong lowering of mood during some months of the year. The results showed higher ratings of subjective wellbeing during the months April to September. Furthermore, the subjects indicated a lowering of mood during the time period from October to February.

### Ratings of Affective State

**Figure 1:**
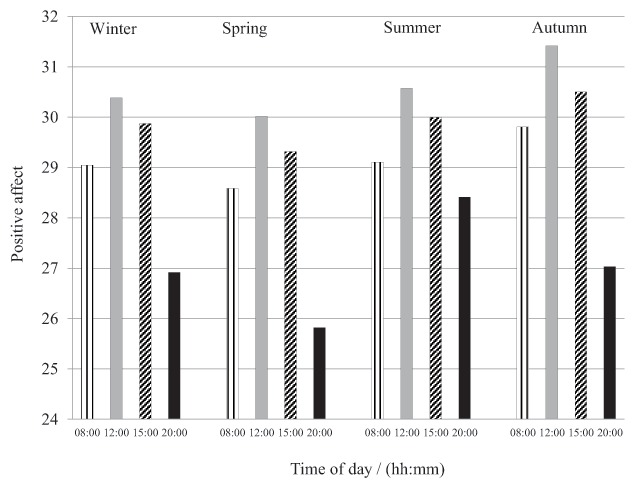
Diurnal and seasonal variation in Positive affect (*N* = 30).

The ratings of Positive affect (PA) showed a diurnal course with an increasing PA from morning to noon and lower values in the evening (F(3,87) = 16.999, *p* < .001). As can be seen in Figure 1, a significant variation (F(3,87) = 4.054, *p* = .010) of the mean daily PA was observed with higher ratings in autumn and summer in comparison to the spring and winter (29.2; 29.0; 27.5; 28.5). See Table 4 for reports of means, medians and standard deviations. Contrasts revealed that the daily average PA during the spring was significantly lower than autumn, F(1,29) = 7.651, *r* = .46, *p* = .01 and summer, F(1,29) = 6.995, *r* = .44, *p* = .013. No significant differences were found between winter and spring or between summer and autumn or between summer and winter.

**Table 4:** Mean, median and standard deviation for ratings of positive affect (PA) at the different times of the day and daily average across the seasons.

Season		Daily average	Time of day/(hh:mm)			
			08:00	12:00	15:00	20:00

Winter	Mean	28.55	28.42	29.72	29.25	26.39
*N* = 30	Median	28.90	28.78	29.67	29.67	27.48
	SD	6.94	7.60	7.59	7.17	6.18

Spring	Mean	27.49	27.65	29.12	28.50	24.85
*N* = 30	Median	26.67	27.89	28.98	28.89	25.76
	SD	6.24	6.68	6.98	6.74	6.36

Summer	Mean	28.97	28.51	29.90	29.36	27.82
*N* = 30	Median	30.05	29.83	30.63	30.44	28.79
	SD	7.18	7.65	7.78	7.72	6.52

Autumn	Mean	29.18	29.15	30.71	29.84	26.54
*N* = 30	Median	30.56	29.50	31.70	30.94	27.60
	SD	7.40	7.71	8.25	8.14	6.41

Furthermore, Figure 1 shows a significant seasonal difference (F(3,87) = 4.609, *p* = .005) in the rating of PA at 20:00, with higher ratings in the summer. Contrasts revealed that the PA in the evening was significantly higher in the summer than during the spring, F(1,29) = 10.76, *r* = .52, *p* = .003, autumn, F(1,29) = 4.94, *r* = .38, *p* = .034, and winter, F(1,29) = 7.78, *r* = .45, *p* = .009.

No other significant seasonal differences were found regarding positive affect.

Moreover, no significant seasonal variation was observed for negative affect (NA). See Table 5 for reports of means, medians and standard deviations.

**Table 5:** Mean, median and standard deviation for ratings of negative affect (NA) at the different times of the day and daily average across the seasons.

Season		Daily average	Time of day/(hh:mm)			
			08:00	12:00	15:00	20:00

Winter	Mean	13.03	12.85	13.17	13.17	12.95
*N* = 30	Median	10.98	11.07	10.97	10.97	11.12
	SD	3.84	3.97	4.02	4.02	3.55

Spring	Mean	12.99	12.84	12.99	12.99	13.15
*N* = 30	Median	12.13	11.78	11.75	11.75	12.11
	SD	2.90	2.94	2.96	2.96	3.01

Summer	Mean	12.81	12.95	12.79	12.79	12.72
*N* = 30	Median	10.82	11.05	10.62	10.62	10.58
	SD	3.50	3.66	3.50	3.50	3.60

Autumn	Mean	12.64	12.68	12.57	12.57	12.72
*N* = 30	Median	11.64	11.5	11.36	11.36	12.17
	SD	3.13	3.16	3.20	3.20	3.14

In order to validate the answers and the relation between retrospective and immediate rating of mood, the results from both instruments were compared. The group that in the retrospective questionnaire reported a history of noticeable or rather strong seasonal variation was compared with the subjects reporting low seasonal variation in mood. Significant differences were found between the two groups showing that the group that retrospectively rated that they were experiencing noticeable or rather strong seasonal variations in mood generally rated their PA lower than the group with no or just a noticeable seasonal variation (F(1,28) = 5.036, *p* = .033). Furthermore, they showed higher average ratings in the summer in comparison to spring, autumn and winter while the seasonal variation in mood was less pronounced in the group experiencing no or less strong symptoms.

### Sleep-Activity Behavior

The subjects typically went to bed between 22:48 and 23:05 and awakened between 06:09 and 06:17 (see Table 6). The data revealed a significant seasonal variation in the sleep-activity behavior (F(3,87) = 3.127, *p* = .030) with later bedtimes in the summer in comparison to the spring, autumn and winter. Contrast analysis showed that during the winter, the subjects slept for a significantly shorter duration than in the summer (F(1,29) = 8.011, *r* = .47, *p* = .008), and the autumn (F(1,29) = 4.983, *r* = .38, *p* = .033).

**Table 6:** Mean, median and standard deviation for wake-up time, bed time and sleep duration across the seasons.

Season		Wake-up time (hh:min)	Sleep time (hh:mm)	Sleep duration (hh:mm)

Winter	Mean	06:17	22:50	07:27
*N* = 30	Median	06:17	23:03	07:15
	SD	00:35	00:38	00:48

Spring	Mean	06:09	22:48	07:27
*N* = 30	Median	06:11	23:01	07:12
	SD	00:23	00:43	00:42

Summer	Mean	06:12	23:05	07:07
*N* = 30	Median	06:15	23:04	07:10
	SD	00:20	00:27	00:27

Autumn	Mean	06:13	22:56	07:16
*N* = 30	Median	06:17	23:03	07:15
	SD	00:27	00:42	00:40

There was no significant difference between spring and winter, spring and summer or between summer and autumn. Furthermore, the wake-up time did not differ between the seasons.

### Subjective Evaluations of the Lighting Conditions in the Offices

Due to the seasonal variation in time of sunrise and sunset the assessments of the lighting conditions were made during the period of daylight illumination in February, April, June, and September. In December, the assessments were completed before sunrise in the morning which meant that the offices were mainly lit by electric lighting.

The analysis of the seasonal ratings of the luminous environment showed that the subjects in general appreciated the lighting conditions in their offices. Annual average ratings of the pleasantness of the light, or hedonic tone, was 4.9. Furthermore, the ratings showed that the light was experienced as fairly strong (4.8) and moderately varied (3.9) with low ratings of flicker (2.1). The subjects reported that the lit environments supported their visual needs (5.5). See Table 7 for reports of means, medians and standard deviations for the different times of assessments.

**Table 7:** Mean, median and standard deviation for assessments of lighting conditions in the offices in the morning and afternoon in February (Feb), April (Apr), June (Jun), September (Sep) and December (Dec).

Parameter		Morning					Afternoon				
	
		Feb	Apr	Jun	Sep	Dec	Feb	Apr	Jun	Sep	Dec

Hedonic tone	Mean	4.86	4.95	4.81	5.15	4.80	4.86	4.99	4.88	5.04	4.73
*N* = 30	Median	4.83	5.00	4.83	5.17	4.69	4.77	5.00	5.00	5.06	4.83
	SD	0.84	0.80	0.82	0.78	0.81	0.82	0.75	0.87	0.92	1.05

Strength	Mean	4.70	5.02	4.74	4.75	4.61	4.80	4.95	4.78	4.80	4.63
*N* = 30	Median	5.00	5.00	4.75	4.84	4.69	4.75	5.00	4.75	4.63	4.75
	SD	1.01	0.52	0.96	0.63	0.98	0.90	0.77	1.01	0.96	1.00

Variation	Mean	3.62	3.94	3.95	3.93	4.00	3.82	3.95	3.95	3.90	4.13
*N* = 30	Median	3.75	4.00	4.00	3.77	4.00	3.81	4.00	4.00	3.77	4.06
	SD	0.56	0.71	0.64	0.75	0.73	0.58	0.66	0.69	0.58	0.75

Flicker	Mean	1.92	2.04	2.41	1.92	2.26	2.12	2.18	2.34	2.14	2.15
*N* = 30	Median	1.00	1.00	2.00	1.00	1.50	2.00	2.00	2.00	1.50	1.50
	SD	1.34	1.58	1.77	1.39	1.57	1.50	1.54	1.74	1.63	1.56

Visibility	Mean	5.79	5.63	5.67	5.71	5.49	5.30	5.28	5.53	5.38	5.32
*N* = 30	Median	6.00	6.00	6.00	6.00	6.00	6.00	6.00	6.00	6.00	6.00
	SD	1.46	1.53	1.20	1.20	1.40	1.56	1.84	1.46	1.55	1.55

Within-subjects contrast was found regarding hedonic tone of the light in the rooms. Contrast analysis showed that the subjects rated the quality of the light in the office higher in the September in comparison to June (F(1,29) = 6.616, *r* = .43, *p* = .015) and December (F(1,29) = 9.734, *r* = .50, *p* = .004).

When separately analyzing the assessments of hedonic tone in the morning and early afternoon a significant seasonal difference was found at 08:00 (F(4,116) = 2.909, *p* = .025) but not at 15:00. Analysis of contrasts showed that in the morning, at 08:00, the subjects rated the hedonic tone of the light in the office higher in September than in February (F(1,29) = 4.227, *r* = .36, *p* = .049), June (F(1,29) = 10.830, *r* = .52, *p* = .003), and December (F(1,29) = 9,298, *r* = .49, *p* = .005). During the afternoon, at 15.00, a significant contrast was found showing higher ratings in September than in December (F(1,29) = 4.966, *r* = .38, *p* = .034).

Regarding visibility in the offices, a significant contrast was found showing that in the afternoon the subjects experienced a higher visibility in June in comparison to February (F(1,29) = 5,585, *r* = .40, *p* = .025).

Furthermore, the contrast analysis showed that the subjects experienced the light in room as less varied in February in comparison to December (F(1,29) = 7.570, *r* = .45, *p* = .010). Additionally, significant differences were found in the afternoon showing higher variation in December in comparison to February (F(1,29) = 5.984, *r* = .41, *p* = .021) and April (F(1,29) = 5.420, *r* = .40, *p* = .027).

Concerning strength of the light in the offices, the contrast analysis showed that the subjects experienced the light in the offices as more strong in April than in February (F(1,29) = 7.580, *r* = .46, *p* = .010) and in December (F(1,29) = 5.080, *r* = .39, *p* = .032). Furthermore, during the morning, the subjects experienced a higher strength of the light in the rooms in April than in December (F(1,29) = 5.764, *r* = .41, *p* = .023), June (F(1,29) = 4.457, *r* = .36, *p* = .043) and September (F(1,29) = 4.874, *r* = .38, *p* = .035).

The experience of the factor flicker showed no significant diurnal or seasonal variation.

### Time Spent Outdoors and Exposure to Bright Daylight

**Figure 2:**
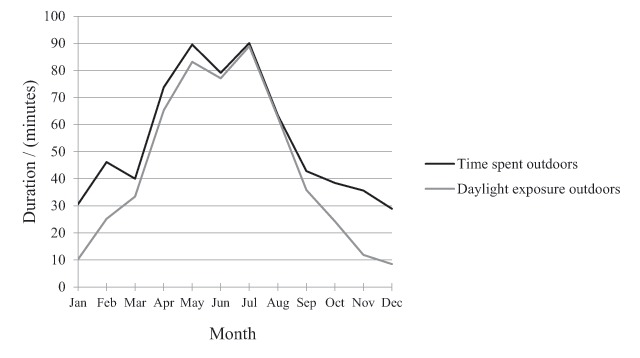
Time spent outdoors and in bright daylight outdoors during the year (*N* = 30).

As can be seen in Figure 2, the weekly diaries revealed a significant seasonal variation (F(3,87) = 29.6, *p* < .001) in the time spent outdoors (i.e. during daylight as well as before sunrise and after sunset). The subjects spent on average 85 minutes outdoors in the summer (May–Jul), 52 minutes in the spring (Feb–Apr), 47 minutes in the autumn (Aug–Oct) and 32 minutes in the winter (Nov–Jan). See Table 8 for reports of means, medians and standard deviations.

**Table 8:** Mean, median and standard deviation for time (minutes) spent outdoors during the different periods of the day across the year.

Season		Time period/(hh:mm–hh:mm)						Daily/minutes
		04:00–08:00	08:00–12:00	12:00–16:00	16:00–20:00	20:00–24:00	00:00–04:00	

Winter	Mean	6.5	2.5	7.2	13.3	2.3	0	31.8
*N* = 30	Median	1.6	0	6.5	12.2	0	0	31.4
	SD	8.0	3.7	7.7	11.2	4.1	0	26.4

Spring	Mean	8.5	3.8	10.6	23.6	4.6	0.5	51.7
*N* = 30	Median	7.5	2.5	9.7	20.0	3.1	0	49.6
	SD	7.7	4.0	8.8	15.8	5.4	2.8	31.7

Summer	Mean	6.8	3.8	12.8	43.3	15.1	0.7	85.1
*N* = 30	Median	3.8	1.7	12.5	42.2	13.3	0.0	81.9
	SD	7.4	6.3	11.8	30.0	12.1	3.6	50.6

Autumn	Mean	6.1	2.1	9.2	23.7	5.5	0	46.6
*N* = 30	Median	3.7	1.3	7.9	17.2	1.5	0	42.3
	SD	6.8	3.1	7.5	22.1	6.7	0.2	35.1

On the other hand, exposure to bright daylight outdoors was on average only 10 minutes during the winter and 41 minutes during spring and autumn while 83 minutes were spent in bright daylight outdoors in the summer (See Figure 2). The exposure to bright daylight outdoors generally occurred before and after work and during the lunch break. See Table 9 for reports of means, medians and standard deviations.

**Table 9:** Mean, median and standard deviation for exposure (minutes) to bright daylight outdoors during the different periods of the day across the year.

Season		Time period/(hh:mm–hh:mm)						Daily/minutes
		04:00–08:00	08:00–12:00	12:00–16:00	16:00–20:00	20:00–24:00	00:00–04:00	

Winter	Mean	0.8	2.1	7.1	0.2	0	0	10.2
*N* = 30	Median	0	0.3	6.5	0	0	0	8.3
	SD	1.5	3.1	7.6	0.6	0	0	10.1

Spring	Mean	7.3	4.1	10.6	19.0	0.4	0	41.4
*N* = 30	Median	5.4	2.5	10.6	16.9	0	0	39.1
	SD	7.0	4.4	8.9	13.2	1.0	0	24.8

Summer	Mean	8.3	4.0	13.2	45.1	12.5	0	83.1
*N* = 30	Median	5.0	1.7	12.8	43.9	12.4	0	80.6
	SD	9.0	6.3	11.8	29.2	9.7	0	47.0

Autumn	Mean	5.8	2.1	9.6	22.3	1.2	0	40.9
*N* = 30	Median	4.4	1.3	7.9	17.3	0	0	35.5
	SD	5.9	3.2	7.8	20.3	2.9	0	31.4

**Figure 3:**
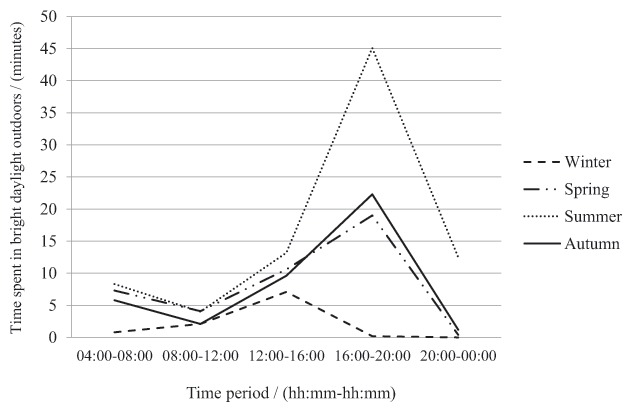
Time spent in bright daylight outdoors during the different time periods of the day across the year (*N* = 30).

Furthermore, the pattern of bright daylight exposure outdoors showed a seasonal difference. The subjects received on average 70% of the daily exposure after 16:00 in the summer. On the other hand, approximately half of the exposure was distributed between wake-up time and 16:00 in the spring (55%) and autumn (45%). The difference between summer, spring and autumn was mainly observed during the time period between 16:00–24:00. During the winter, all the bright daylight exposure outdoors occurred between wake-up time and 16:00. Figure 3 shows that significant seasonal differences were observed during the time periods between 04:00–08:00 (F(3,87) = 13.810, *p* < .001), 08:00–12:00 (F(3,87) = 3.343, *p* = .023), 12:00–16:00 (F(3,87) = 6.405, *p* = .001), 16:00–20:00 (F(3,87) = 42.868, *p* < .001) and 20:00–00:00 (F(3,87) = 42.974, *p* < .001).

Analysis of contrasts showed that during the winter, the subjects were exposed to significantly lower amounts of bright daylight outdoors in the early morning, between 04:00 and 08:00, than in the autumn (F(1,29) = 31.893, *r* = .72, *p* = < .001), summer (F(1,29) = 24.174, *r* = .67, *p* = < .001) and spring (F(1,29) = 27.464, *r* = .70, *p* = < .001). Moreover, during the summer the subjects were exposed to significantly higher amounts of bright daylight outdoors in the morning, between 04:00 and 08:00, than in the autumn (F(1,29) = 5.042, *r* = .38, *p* = .033). However, no significant differences in exposure were found concerning comparisons between summer and spring and between spring and autumn.

During the winter, the subjects were exposed to significantly lower amounts of bright daylight outdoors in the morning, between 08:00 and 12:00 than in the spring (F(1,29) = 6.832, *r* = .43, *p* = .014). Moreover, during the spring the subjects received more bright daylight outdoors than in the autumn (F(1,29) = 8.663, *r* = .48, *p* = .006). There were no significant differences between summer and spring and between summer and autumn.

Regarding the time period between 12:00 and 16:00, contrast analysis revealed that during the winter the subjects were exposed to significantly lower amounts of bright daylight outdoors than in the spring (F(1,29) = 17.735, *r* = .61, *p* < .001), summer (F(1,29) = 17.089, *r* = .61, *p* < .001) and autumn (F(1,29) = 5.483, *r* = .40, *p* = .026). There were no significant differences between summer, spring and autumn.

During the winter the subjects were exposed to significantly lower amounts of bright daylight outdoors in the evening, between 16:00 and 20:00 than in the spring (F(1,29) = 61.628, *r* = .82, *p* < .001), summer (F(1,29) = 72.067, *r* = .84, *p* < .001) and autumn (F(1,29) = 35.540, *r* = .74, *p* < .001). Furthermore, during the summer the subjects were exposed to significantly higher amounts of bright daylight outdoors than in the spring (F(1,29) = 35.881, *r* = .74, *p* < .001) and autumn (F(1,29) = 26.694, *r* = .69, *p* < .001). There were no significant differences between spring and autumn for the time period between 16:00 and 20:00.

Concerning the evening, between 20:00 and 24:00, during the winter the subjects were exposed to significantly lower amounts of bright daylight outdoors in comparison to the spring (F(1,29) = 6.513, *r* = .42, *p* = .016), summer (F(1,29) = 49.933, *r* = .80, *p* < .001), and autumn (F(1,29) = 5.219, *r* = .39 *p* = .030). Moreover, during the summer the subjects were exposed to significantly higher amounts of bright daylight outdoors in comparison to the spring (F(1,29) = 46.865, *r* = .79, *p* < .001) and autumn (F(1,29) = 39.399, *r* = .76, *p* < .001). There were no significant differences between spring and autumn.

Table 10 shows seasonal comparisons of photoperiod, sleep-wake behavior and work schedules. Regarding the length of the photoperiod, there is a considerable seasonal variation on this latitude (56°N) with daylight during more than nineteen hours at the summer solstice and less than seven hours at the winter solstice. This results in quite short photoperiods during a part of the year but on the other hand long days during a substantial period of the year.

**Table 10:** Time of sunrise and sunset, work schedules, sleep-wake behavior and daylight availability during leisure time (*N* = 30).

Month	Sunrise (hh:mm)	Sunset (hh:mm)	Start work (hh:mm)	End work (hh:mm)	Wake up time (hh:mm)	Bedtime (hh:mm)	Time in bed (hh:mm)	Start work-Sunrise (hh:mm)	End work-Sunset (hh:mm)	Daylight hours during leisure time (hh:mm)

Jun	04:24	20:58	07:48	16:24	06:08	23:03	07:06	03:24	04:34	07:58
Jul	04:46	20:45	07:50	16:19	06:14	23:11	07:04	03:04	04:26	07:30
Aug	05:41	19:46	07:51	15:55	06:11	22:57	07:14	02:10	03:51	06:01
Sep	06:41	18:26	07:48	16:40	06:14	23:00	07:18	01:07	01:46	02:53
Oct	07:36	17:08	07:48	16:28	06:14	22:55	07:18	00:12	00:40	00:52
Nov	07:46	16:01	07:59	16:23	06:17	22:45	07:30	00:13	00:00	00:13
Dec	08:33	15:34	07:57	16:32	06:15	22:52	07:21	00:00	00:00	00:00
Jan	08:28	16:06	07:50	16:49	06:18	22:50	07:27	00:00	00:00	00:00
Feb	07:36	17:11	07:45	16:54	06:06	22:49	07:13	00:09	00:17	00:26
Mar	06:28	18:13	07:50	16:53	06:06	22:39	07:26	01:22	01:20	02:42
Apr	06:03	19:17	07:54	16:28	06:15	22:53	07:21	01:51	02:49	04:40
May	04:56	20:17	07:52	16:27	06:14	23:00	07:13	02:56	03:50	06:46

### Interrelationships Between the Different Parameters

The analysis of Pearson correlation coefficients showed no significant relationship between how much time that was spent outdoors in bright daylight and the daily average ratings of positive affect, neither across the year nor during the different seasons. Furthermore, there was no direct relationship between the total daily amount of exposure to bright daylight outdoors and ratings of positive affect in the evening.

The correlation analysis showed a significant relationship between exposure to bright daylight outdoors in the evening, between 20:00 and 24:00, and bed time (*r* = 0,211, *p* = .010, 1-tailed). In addition, the analysis of Pearson correlation coefficients showed a significant relationship between the length of the photoperiod and bed time (*r* = 0,154, *p* = .046, 1-tailed).

Across the year, no significant relationship was found between the parameters comprising the assessments of lighting conditions in the offices and ratings of daily average positive affect or ratings of positive affect in the afternoon. However, additional analyses for the five times of assessment were performed in order to investigate if there were any correlations between the perception of the light environment and positive affect during certain periods of the year. The analysis showed a significant negative correlation between the perception of the strength of the light in the room and positive affect (*r* = –.249, *p* = .028, 1-tailed) in December. Also, there was a significant relationship between how varied the light in the room was perceived in June and positive affect (*r* = .245, *p* = .03, 1-tailed). Furthermore, a significant correlation was found between the assessments of flicker and positive affect in April (*r* = –.240, *p* = .035, 1-tailed).

The analysis showed a significant correlation between the length of the photoperiod and how much the subjects were exposed to bright daylight outdoors during the day (*r* = .606, *p* < 0.001, 1-tailed) and during the evening 20:00–24:00 (*r* = .628, *p* < 0.001, 1-tailed).

## Discussion

The study investigated seasonal variation in mood among a group of Swedish office workers. In line with results from other studies, the ratings of positive affect (PA) showed a diurnal variation with an increase from morning to midday and a decline from early afternoon to the evening [46,71,72].

First, the retrospective ratings of seasonality showed that most of the participants experienced a higher subjective wellbeing during the summer and many experienced lower mood during the winter. This was confirmed in the results from the measurements that were monthly repeated across the year showing a significant seasonal variation in overall mood with higher ratings during the summer and autumn than during the spring and winter. The seasonal difference in mood ratings were attributed to higher values in the evening. This may be interpreted as a consequence of more light exposure during the day and especially in the afternoon and evening resulting in a delayed sleep-wake rhythm or an acute alerting effect of more bright light exposure. However, there were no seasonal differences in the ratings during the other times of the day which should have been noticed as a result of a delayed circadian rhythm [46].

Furthermore, there was no direct relationship between the ratings of positive affect and the amount of bright daylight exposure outdoors which has been shown in previous studies [43]. However, the results are in line with findings reported in other field studies [44,46].

The sleep-activity behavior of the participants in relation to the natural photoperiod reveal that during a substantial period of the year the wake-up times occurred before sunrise and during the winter many people started working before the sunrise and ended work after sunset, commuting to and from work when it was dark. This explains the difference between time spent outdoors and time spent outdoors in bright daylight during the winter. This also indicate that the occupation and work schedules only permitted exposure to bright daylight outdoors during the work breaks in the winter months of December and January and only for a short time before and after work in October, November and February. The results confirm findings from other studies, showing that many people, of different ages and living at higher latitudes of the northern hemisphere, spend most of their time in environments with indoor light intensities [39,40,73]. Moreover, this longitudinal study shows that the subjects spent nearly one hour, or 2.5 times, more outdoors in the summer in comparison to the winter and approximately 1.5 times more outdoors in spring and autumn in comparison to the winter. The seasonal variation in the time spent in bright daylight outdoors was even more pronounced. The amount of exposure to bright daylight outdoors showed seasonal differences with a larger amount in the summer in comparison to spring, autumn and winter, especially in the afternoon and evening. The results showed that exposure to bright daylight outdoors increased from spring reaching highest values in the summer then beginning to decrease in autumn and reaching lowest values in winter. During the winter months, the subjects spent on average only 10 minutes per day in bright daylight outdoors. In the summer, they spent approximately eight times more of their time in bright daylight outdoors and four times more in the spring and autumn compared with the winter. Other studies, carried out at latitudes between 30–45°N have reported bright light exposures of 30 minutes in the winter and 90–230 minutes during the summer [39,40,49,74]. The exposure to bright daylight outdoors typically occurred before and after work and during the lunch break.

Additionally, the results showed that the subjects mainly were exposed to a combination of daylight and artificial light from linear and compact fluorescent tubes in the workplace and primarily light radiation from incandescent lamps in the early morning and afternoon/evening during the winter and parts of the spring and autumn. In the summer, the subjects were to a larger extent exposed to daylight outside during the leisure time. In accordance with other studies, the results suggest that the participants were not only exposed to more short-wavelength light radiation in the summer but also may have experienced a seasonal variation in spectral composition of the light exposure, with higher relative contribution of short-wavelength light radiation, especially in morning and evening, in the summer compared with the winter, spring and autumn [8,41]. This indicate that the radiant exposure during the summer may be more powerful in effecting the human physiology, neuroendocrine function and in entraining and aligning the circadian rhythms in comparison to the winter, spring and autumn. On the other hand, results from studies investigating the influence of light history have shown an increased sensitivity to light radiation following exposure to light of low intensities and darkness [75,76,77]. Moreover, bistable properties of the intrinsically photoreceptive retinal ganglion cells (ipRGCs) possibly have an impact on the non-image forming effects of the radiant exposure [78].

A seasonal difference in bed times, but not wake up times, was observed indicating shorter sleep durations in the summer. These results are to some extent supported by earlier findings [8,57,58]. Then again, Park et al. [3] did not find a seasonal difference in sleep length and Figueiro and Rea [38] found longer sleep durations as well as higher sleep efficiency and shorter sleep latencies in the summer than in the winter. In another longitudinal field study conducted in Sweden, also investigating seasonal variations in various sleep parameters, a seasonal variation concerning bedtime was found although no seasonal changes were found regarding sleep duration [56]. As mentioned earlier, the inconsistencies between the results may be explained by differences regarding study design, location where the study was carried out, number of participants and instruments used for data collection. Therefore, further studies are needed in order to increase the knowledge of seasonal variation in different sleep characteristics and the relationship between light exposure patterns and sleep parameters.

Turning to the results from the seasonal assessments of the experienced lighting conditions, the subjects rated the pleasantness of the light higher in September compared to the assessments in June and December. This was attributed to higher ratings in the morning rather than in the early afternoon. A larger availability of daylight in June was hypothesized to result in higher ratings during this time of measurement as several studies have showed that people prefer day lit environments over artificial lighting [60,61]. However, the subjects could control the daylight with venetian blinds. This could have resulted in that more daylight not necessarily entered the offices during the summer. In the present study, more than half of the offices had windows facing NE-W which may have influenced the daylight availability and its distribution in the room due to direct sunlight through the windows and use of venetian blinds. This may also explain why the light in the offices not was experienced as stronger in the summer. A higher sensitivity to light in the winter and higher light levels outdoors in the summer may also explain the results [77,79]. Other studies have found a relationship between the experience of the lit environment and psychological well-being [36]. In this study, no correlations were found across the year but a relationship between parameters included in the assessments of the lighting conditions and the ratings of positive affect was found during some time periods of the year which motivates further studies investigating relationships between qualities of the light environment in the work place and psychological well-being. Also, the analyses did not consider the weather conditions during the times for assessments which may have had an effect on the results and should be closer investigated in future studies.

The present study investigated the seasonal variation in mood, behavior and light exposure during a typical workday. Other studies have shown that light exposure between weekdays and weekends varies [44,80]. Self-report diaries have been used in other field studies in order to estimate the light exposure of people in their every-day life [45,46]. Furthermore, other researchers have shown self-report diaries to be a useful and reliable instrument when investigating use of lighting controls and energy-related behavior in the home environment [81,82]. In this study, bright daylight exposure outdoors and sleep-activity data were collected from diaries which may have an influence on the reliability of the data. Ambulatory instruments, with higher resolution of the collected data, possibly had resulted in a higher reliability of the measurements [39,56].

There was on average a one-degree variation in ambient room temperature across the year which is a small variation and it seems unlikely that it would have affected the seasonal variations in mood and behavior that were found in the study (see Table 2). Highest mean ambient room temperatures were measured in June/July (22.7°C) and lowest values were recorded in September/October (21.7°C). According to Swedish recommendations the ambient room temperature should be within the range 20–24°C during the winter and within the range 20–26°C during the summer [83].

The strength of the present study is the longitudinal within subject design with data regarding sleep-activity behavior, bright daylight exposure outside and psychological wellbeing collected during three days every month across the year. Also, the perception of the lighting conditions in the working environments was assessed at different seasons and at different times of the day.

## Conclusion

In conclusion, a seasonal variation in mood and sleep-activity pattern was observed. The study confirmed results from earlier studies investigating light exposure in real working and living environments, carried out at latitudes between 30–45°N, showing that many people spend most of their time indoors receiving bright light for relatively short periods during particularly winter but also during spring, summer and autumn [40,73]. Furthermore, the group of Swedish office workers seems to be exposed to even less bright light during the winter in comparison to people living on more southern latitudes, both as a consequence of less time spent outdoors but also by the length of the photoperiod [40,49,74]. This suggests that there may be a lack of exposure to short-wavelength light during the time that office working people in Sweden spend indoors which illustrates the importance of the indoor environment for modern-day people [38]. Consequently, this indicates that it is crucial to designing suitable lighting conditions in the workplaces and developing an appropriate lighting technology for the home environment.

There are several ways of replacing a possible lack of exposure to short-wavelength light during the substantial amount of the daytime that modern-day people in Sweden spend indoors. One way to achieve this is to determine that the office space is well day lit [84]. Another way is by using light sources enriched in the short-wavelength part of the spectrum. Vetter et al. [85] demonstrated that lighting from luminaires equipped with blue-enriched light sources had a significant effect on the entrainment of the circadian rhythm of sleep and behavior. Other studies have shown positive effects in alertness, mood, evening fatigue, irritability, concentration, and eye discomfort as well as in vitality and mental health [86,87]. However, Iskra-Golec et al. [88] found that, although blue-enriched light of 17000 K in comparison to light from light sources with a correlated color temperature of 4000 K had an activating effect, especially in the morning but also when considered over the whole day, the measurements of mood showed a declining trend during the course of the day and was slightly lower in the blue-enriched condition. Interestingly, they also found that the lighting from blue-enriched light sources was experienced as more bright in the morning and afternoon but not at other times of the day which demonstrates the importance of also recognizing daily variations concerning the impact on visual aspects of the lit environment.

Finally, light radiation and the lit environment have a major influence on human physiology, psychology and health-related quality of life in addition to work performance and work satisfaction [11,35]. New solid-state light sources are increasing in use, mainly for energy-efficiency reasons, and will be in use for a very long time. For this reason, it is important to ensure that the properties of these light sources support the physiological and psychological needs of occupants. More laboratory studies and field studies conducted in real working and living environments are needed in order to define the characteristics of light environments that support the physiological, psychological and visually ergonomic needs of different groups of human users.
